# Life-threatening hyponatremia due to intravenous n-acetylcysteine treatment in an infant: a case report

**DOI:** 10.4076/1757-1626-2-8347

**Published:** 2009-09-01

**Authors:** Juan Mayordomo-Colunga, Elene Larrea, Mónica García, Sonsoles Suárez, Julián Rodríguez

**Affiliations:** Paediatric Emergency Unit, Department of Pediatrics, Hospital Universitario Central de AsturiasOviedoSpain

## Abstract

**Introduction:**

N-acetylcysteine has proven to be effective in paracetamol intoxications, but there is no consensus regarding its way of administration. Here, we report a case to highlight the importance of careful management of intravenous n-acetylcysteine.

**Case presentation:**

A two-month old infant was seen in our paediatric emergency department due to paracetamol poisoning after repeated supratherapeutic doses. She was treated with intravenous n-acetylcysteine diluted with dextrose 5%, according to the 20-hour standard protocol. Eight hours later she developed two tonic-clonic seizures and was subsequently intubated. By that time, she had received almost 1 liter of 5% dextrose, and serum sodium was 114 mg/dL. A rapid correction was done with hypertonic saline and the child experienced a good outcome, without any sequelae.

**Conclusion:**

Intravenous n-acetylcysteine administration must be done carefully. Amount of liquid administrated and sodium monitoring should be kept in mind, with special care in small children.

## Introduction

Paracetamol is a worldwide-used, safe and effective antipyretic and analgesic for children when used at therapeutic doses. It is nowadays the leading cause of drug poisoning among young children [1]. Repeated supratherapeutic doses with non self-harming intent is an increasing type of paracetamol intoxication, both in adult and paediatric patients [2].

This medication achieves its highest serum levels about four hours after ingestion [3]. Serum paracetamol level determination should be performed at that time if ingested amount is over 150 to 200 mg/kg in a single dose [4,5]. If the value is above the study line of Rumack-Matthew nomogram [6], N-acetylcysteine (NAC) must be administered, either intravenously or orally. Activated charcoal is useful to lower intestinal absorption when ingestion takes place within the previous 60 to 90 minutes [3,7].

Here, we present a case, which derived in a life-threatening condition in an infant due to intravenous administration of NAC. We also discuss the use of NAC so as to avoid complications in small children.

## Case presentation

A two-month old Caucasian Spanish infant was seen in our pediatric emergency department (PED) because of paracetamol overdosing. She was febrile due to vaccination and her parents gave her three consecutive doses of 500 mg instead of 50 mg of paracetamol (weight 4.7 kg), separated by 6 hours. The infant was brought to our PED two hours after the last dose.

Plasma paracetamol level was determined on arrival (113.5 µg/mL). The child was admitted to the ward and intravenous NAC infusion was started following the standard 20-hour protocol as paracetamol level was high (Table 1).

**Table 1.** Blood determinations at admission (PED), at the time seizures developed (WARD) and after rapid correction with hypertonic saline in the pediatric intensive care unit. PED: pediatric emergency department; PICU: pediatric intensive care unit

	PED	WARD	PICU
Glucose (mg/dl)	117	290	137
Sodium (mg/dl)	134	114	124
AST (U/l)	64	57	61
ALT (U/l)	39	36	38
Serum paracetamol level (µg/mL)	113.5	17.8	7.8

Eight hours later, the child presented a 2-minutes generalized seizure which responded to intravenous diazepam. Fifteen minutes later, she had another tonic-clonic seizure which also responded to diazepam after 6 minutes. Due to impaired consciousness and suspicion of liver encephalopathy she was intubated and transferred to the pediatric intensive care unit. Blood analysis showed hyponatremia of 114 mEq/L, which was managed with a rapid correction of hypertonic saline until the serum sodium reached 124 mmol/l. Then, sodium was gradually increased via isotonic saline and furosemide boluses. Paracetamol levels were undetectable by 15 hours after admission (Table 1).

The girl was extubated ten hours later, and evoluted satisfactorily. She was followed up as an outpatient during the following months, and her neurological status was completely normal.

## Discussion

Paracetamol poisoning is the main cause of acute liver failure in adult patients in the West World. In children, paracetamol has fewer toxic effects, but due to its wide use, it remains a concern nowadays. The risk of hepatoxicity is higher in repeated supratherapeutic doses administration than in single overdose [8]. A frequent mistake in pediatric dose administration is made when the parents miscalculate the appropriate dose or when there is confusion with paracetamol concentration due to different presentations of the same product [9]. In our case, dosage was ten times higher than the right one [10].

NAC is useful as an antidote for paracetamol overdosing as it increases gluthatione, which binds and inactivates the hepatotoxic metabolite N-acetyl-p-benzoquinoneimine. It is indicated when paracetamol plasma concentration is above the study line of Rumack-Matthew nomogram in single overdoses [6]. If time of ingestion is not known or if the patient received repeated supratherapeutic doses, correct risk-stratification cannot be done with the use of Rumack-Matthew nomogram. In those cases, it seems prudent to administer NAC if serum paracetamol concentration is over 20 µg/ml [11]. Moreover, delayed NAC administration has been identified as one of the risk factors to a worse outcome [12]. According to this, the decision of prescribing NAC to our child was correct.

There is no consensus regarding NAC administration as it seems to be effective if used both intravenously and orally [7,12,13]. Some advantages for intravenous administration are that it can be used in patients at risk of impaired consciousness and its shorter duration when compared to oral one. Oral dose of n-acetylcysteine involves a large volume and would greatly increase the risk of regurgitation and aspiration, mostly if liver failure develops which could impair conscious level.

Protocols as the 20-hour one used in our case must be abandoned in small patients for intravenous NAC administration. According to them, patients are given a loading dose of 150 mg/kg of NAC in 40 to 200 ml of 5% dextrose over 15 minutes, followed by 50 mg/kg in 500 ml of 5% dextrose over 4 hours and then 100 mg/kg in 1000 ml of 5% dextrose over the next 16 hours. By eight hours of treatment, our child had received 950 mL of 5% dextrose. If the protocol would have been completed, she would have received up to 361.7 ml/kg of free water. Therefore, NAC concentration should be of 40 mg of NAC per ml of 5% dextrose [11,14] or drug information sheet for patients weighting 10 kg should be followed (Acetadote^®^). This should avoid complications as cerebral edema or hyponatremia [14].

In conclusion, intravenous NAC administration must be done carefully. Amount of liquid administrated and sodium monitoring should be kept in mind, with special care in small children.
